# The Progress of Investigating the CD137-CD137L Axis as a Potential Target for Systemic Lupus Erythematosus

**DOI:** 10.3390/cells8091044

**Published:** 2019-09-06

**Authors:** Anselm Mak, Herbert Schwarz

**Affiliations:** 1 Department of Medicine, Yong Loo Lin School of Medicine, National University of Singapore, Singapore 119228, Singapore; 2 Department of Physiology, Yong Loo Lin School of Medicine, National University of Singapore, Singapore 117593, Singapore; 3 Division of Rheumatology, University Medicine Cluster, National University Health System, Singapore 119228, Singapore; 4 Immunology Programme, National University of Singapore, Singapore 117456, Singapore

**Keywords:** CD137, CD137 ligand, lupus, renal, cerebral, SLE

## Abstract

Costimulatory molecules facilitate cross-talks among leukocytes via mutual stimulatory and inhibitory signalling, contributing to diverse immunological outcomes in normal physiological responses and pathological conditions. Systemic lupus erythematosus (SLE) is a complex multi-systemic autoimmune condition in which cellular communication through the involvement of costimulatory molecules is crucial in driving proinflammatory responses from the stage of autoantigen presentation to the subsequent process of pathogenic autoantibody production. While the physiology of the costimulatory systems including OX40-OX40L, CD28/CTLA-4-CD80/86, ICOS-B7RP1 and CD70-CD27 has been relatively well studied in SLE, recent data on the immunopathology of the CD137-CD137 ligand (CD137L) system in murine lupus models and patients with SLE highlight the critical role of this costimulatory system in initiating and perpetuating the diverse clinical and serological phenotypes of SLE. CD137, a membrane-bound receptor which belongs to the tumour necrosis factor receptor superfamily, is mainly expressed on activated T cells. Activation of the CD137 receptor via its interaction with CD137L which is expressed on antigen present cells (APC) including B cells, triggers bi-directional signalling; that is, signalling through CD137 as well as signalling through CD137L (reverse signalling), which further activates T cells and polarizes them to the Th1/Tc1 pathway. Further, via reverse CD137L signalling it enhances differentiation and maturation of the APC, particularly of dendritic cells, which subsequently drive proinflammatory cytokine production. In this review, recent data including our experience in the manipulation of CD137L signalling pertaining to the pathophysiology of SLE will be critically reviewed. More in-depth understanding of the biology of the CD137-CD137L co-stimulation system opens an opportunity to identify new prognostic biomarkers and the design of novel therapeutic approaches for advancing the management of SLE.

## 1. The Molecular Structure and Physiology of CD137 and CD137 Ligand

### 1.1. Genetics and Molecular Structure of CD137 and CD137 Ligand

The human *CD137* (also named *4-1BB* or *TNFRSF9*) gene is located on chromosome 1p36 and shares the gene location with a cluster of related genes encoding for other tumour necrosis factor receptors in the superfamily, (*TNFRSF*) including *OX-40, CD30* and *TNFRII* [1,2]. Similar to other members of the TNFRSF, the membrane-bound CD137 comprises extracellular domains characterized by conserved cysteine-rich repeats, a transmembrane domain comprising glycoproteins and a cytoplasmic domain which lacks a signal transduction motif [3]. As in most of the TNFRSF members, CD137 exists in a membrane-bound and a soluble form. The soluble form of CD137 (sCD137) is produced by differential splicing, in contrast to other soluble forms of the TNFRSF members including CD27, CD30, CD40, CD95 and CD120a which are released by proteolytic cleavage. Functionally, sCD137 binds to CD137 ligand (CD137L) (also named 4-1BBL or TNFSF9) and abrogates ligand-mediated activities such as T cell proliferation and Interleukin (IL)-2 secretion, serving as a negative regulator that antagonizes the costimulatory activities of membrane-bound CD137 [4,5,6,7]. CD137L is a transmembrane polypeptide which belongs to the TNF ligand superfamily. The gene which encodes CD137L in human locates on chromosome 19p13.3 [8] where other genes belonging to the same TNF ligand family including *CD40L* and *CD27L* are clustered. Just as CD137, CD137L also exists in a membrane-bound and a soluble form.

### 1.2. Immunological Alterations and Downstream Signalling of CD137 and CD137L

An experiment in the late 1980s demonstrated that concanavalin A (ConA) activation of murine CD8^+^ and CD4^+^ T cells up-regulated *CD137* gene expression [9]. Later in 1993, using T-cell leukaemia virus type 1-transformed human T cells, the human homologue of the murine *CD137* gene sequence was cloned [1]. In both the murine and the human systems, CD137 is predominantly expressed on CD8^+^ T cells (and to a lesser extent on CD4^+^ T cells), peaking 48 h after in vitro stimulation [10]. Conversely, treatment with cyclosporin A, a potent T cell inhibitor which antagonizes the function of calcineurin [11], was shown to suppress the expression of CD137 mRNA in both CD4^+^ and CD8^+^ T cells [9], confirming that CD137 expression is enhanced and suppressed upon T cells activation and suppression, respectively.

Upon ligation with CD137L which is expressed on antigen-presenting cells (APCs) including B cells, bi-directional signalling ensues; that is, signalling through CD137 as well as signalling through CD137L (reverse signalling), initiating the stimulation of the two interacting cells in a CD28-independent manner [12,13,14,15,16]. Such observation suggests that CD137 ligation initially enhances and subsequently brakes pro-inflammatory immune responses, aiming to function as a critical immune checkpoint to prevent the immune system from overwhelmed stimulation that may potentially harm the biological system [11].

**Figure 1:**
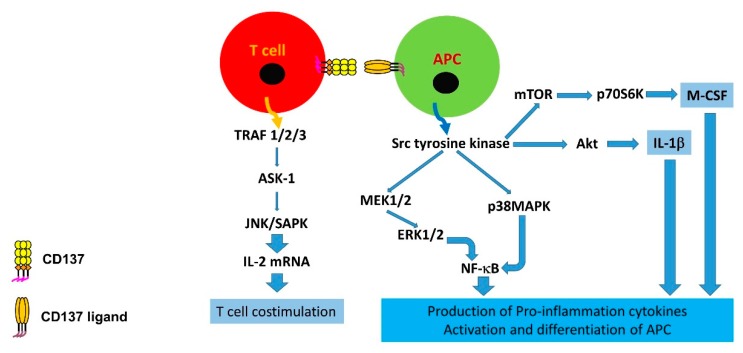
Signalling pathways mediating signal transduction of CD137 and CD137L in human leukocytes. APC: Antigen-presenting cell.

A number of intracellular molecules involved in various signalling pathways are triggered when the CD137-CD137L system is activated. On the CD137 side, similar to the signalling mechanisms which are activated by other members of the TNFRSF, engagement by CD137L triggers signalling of TRAF (TNF receptor-associated factor) 1, TRAF2 and TRAF3. TRAF2 activation is required to activate JNK (c-Jun *N*-terminal kinase)/SAPK (stress-activated kinase) as a result of ASK-1 (Apoptosis signal-regulating kinase 1) stimulation [17]. Activation of JNK/SAPK subsequently enhances the transcription of Interleukin (IL)-2 mRNA, a critical factor for T cell proliferation and survival. Upon activation of CD137L in human monocytes, NF-κB (nuclear factor-kappa-light-chain-enhancer of activated B) and a number of protein tyrosine kinases downstream including p38 MAPK (mitogen-activated protein kinase), MEK (mitogen-activated protein kinase kinase), ERK1/2 (extracellular signal-regulated kinases) and PI3K (phosphoinositol 3-kinase; PI: phosphatidylinositol) are induced, leading to pro-inflammatory cytokine expression [18]. Activation of the Src family tyrosine kinase/Akt (serine/threonine kinase) pathways enhances IL-1β production while activation through the Src family tyrosine kinase/mTOR (mechanistic target of rapamycin)/p70S6K promotes cell viability and increases M-CSF production [15,19,20]. Figure 1 denotes the major signalling pathways involved in the human CD137-CD137L co-stimulation system.

CD137L signalling in human monocytes induces their differentiation to proinflammatory dendritic cells (DCs) [20,21,22,23,24]. CD137L signalling in murine monocytes induces their differentiation to cells that seem to be tolerogenic in nature [24]. The basis of this species difference between murine and human CD137L signalling is likely the pronounced differences in the structure of murine and human CD137L. While for most members of the TNFSF the homology between the human and murine protein is 60–80%, CD137L is only conserved to 37% at the amino acid level between man and mouse [8].

## 2. CD137 and CD137L in Various Autoimmune Conditions

The interaction between CD137 and CD137L has been implicated in various autoimmune conditions of which collagen-induced arthritis (CIA) [25], experimental autoimmune uveoretinitis (EAU) [26] and experimental autoimmune encephalitis (EAE) [27] have been most investigated. The CIA mouse model is characterized by Th17-driven chronic inflammatory arthritis in DBA/1 mice [25,28]. Stimulation of CD137 by agonistic anti-CD137 antibody polarizes the T cells towards a Th1/Tc1 response [29] thereby weakening the pathogenic Th17 polarization and ameliorating the severity of arthritis in CIA mice [25]. Stimulation of CD137 drives the expansion of CD8^+^ T cells and CD8^+^CD11c^+^ cells which are rich sources of IFN-γ, the signature cytokine for a Th1/Tc1 response [30,31]. As in the CIA model, agonistic CD137 antibodies alleviate and prevent EAU by similar mechanisms as CD137 stimulation works to relieve chronic arthritis in CIA mice [25]. The examples illustrated in CIA and EAU mice underscore the importance of the integrity of the CD137 signalling system in mitigating the respective Th17-driven autoimmune conditions through activation and enhancement of CD8^+^ T cells which potently produce IFN-γ, one of the cytokines which is involved in suppressing chronic Th2- and Th17-dominated inflammation (see Section 3.1 and Section 3.2 for the proposed mechanisms).

Along with Th-1-driven autoimmune diseases, it is tempting to investigate the impact of CD137-CD137L system manipulation on autoimmune conditions chiefly driven by the Th17 axis such as EAE and systemic lupus erythematosus (SLE). In EAE, apart from the confirmation that CD137 stimulation activates CD8^+^ T cells and increases IFN-γ that leads to reduction in EAE severity, an IFN-γ independent pathway is operative [27]. It was demonstrated that in IFN-γ-deficient EAE mice, CD137 stimulation expanded regulatory T cells (CD4^+^CD25^+^FoxP3^+^) (Tregs) and reduced Th17 cells through the reduction of IL-6 production by pathogenic CD4^+^ T cells reactive to MOG_35–55,_ an inducer of EAE in GKO and GRKO B6 mice [27]. As a result of expansion and activation of Tregs and suppression of Th17 cells, the resultant reduction in pro-inflammatory signals reduces the severity of EAE without the participation of IFN-γ after CD137 stimulation [27].

## 3. CD137 and Systemic Lupus Erythematosus

### 3.1. Animal Studies

At the time of writing of this review, there was one study which explored the role of CD137 in SLE by knocking out CD137 receptor in MRL-Fas^lpr^ lupus-prone mice [32] and two studies which demonstrated the protective effects against SLE by stimulating CD137 in two different lupus-prone mouse models [33,34]. Compared to MRL-Fas^lpr^ mice, CD137-deficient MRL-Fas^lpr^ mice exhibited more severe SLE phenotype including pronounced splenomegaly, lymphadenopathy, dermatitis and glomerulonephritis, in addition to a significantly higher 5-month mortality rate than the MRL-Fas^lpr^ mice (80% versus 40%), with renal inflammation being the main cause of death [32]. Further mechanistic analyses revealed that CD137-deficient MRL-Fas^lpr^ mice had a higher number of CD4^+^ T cells and double-negative T cells (DNTC) while numbers of CD8^+^ T cells were not altered [32]. Furthermore, CD137-deficient MRL-Fas^lpr^ mice showed increased germinal centre formation associated with a trend of higher proportions of functional B220^+^CD5^-^ B cell, coupled with higher serum anti-nuclear IgG1/2a antibody, total immunoglobulin and anti-dsDNA antibody levels at the age of 3 months as compared to the MRL-Fas^lpr^ mice [32]. Histological studies demonstrated increased glomerular infiltrates and renal deposition of IgG and C3 in CD137-deficient MRL-Fas^lpr^ mice [32]. The opposite effect is obtained when CD137 is activated by the agonistic anti-CD137 antibody. In that case, lupus manifestations are alleviated as demonstrated in two studies using the B6.lpr [33] and NZB x NZW F_1_ mouse models [34]. In the study by Foell et al., three i.p. injections of agonistic anti-CD137 antibody given to NZB x NZW F_1_ lupus-prone mice between the ages of 26 and 35 weeks reversed acute SLE, abrogated chronic disease and lengthened the survival of the mice from the mean of 10 months to more than 2 years [34]. Alleviation of SLE disease manifestation is postulated to be due to reduced IgG anti-dsDNA antibody production as a result of anergy of autoreactive CD4^+^ T cells and an increase in Treg cells. In an attempt to understand whether agonistic anti-CD137 antibody could delay the onset of SLE development, 8-week old NZB x NZW F_1_ mice were treated with one i.p. injection of agonistic anti-CD137 antibody prior to the development of SLE phenotype and the appearance of serum anti-dsDNA antibodies. Despite the short half-life of agonistic anti-CD137 Ab of only around 7 days, the young pre-disease mice treated with anti-CD137 antibody continued to survive without emergence of proteinuria, coupled with a suppression of anti-dsDNA IgG until the mice reached 30 weeks of age. Apart from delaying the onset of SLE, treatment with one dose of agonistic anti-CD137 antibody in diseased NZB x NZW F_1_ mice over the age 36 weeks led to marked reduction of serum anti-dsDNA IgG and milder renal inflammation compared to age-matched untreated or isotype-matched antibody-treated mice [34]. A similar study involving treatment of B6.lpr or MRL^lpr^ lupus-prone mice with agonistic anti-CD137 antibody (clone 2A) i.p. for three weekly doses commencing at the age of 2–3 months produced similar results in terms of improvement of survival, reduced severity of cutaneous inflammation, splenomegaly, lymphadenopathy and lupus glomerulonephritis and a reduction in serum anti-dsDNA IgG levels compared to mice of the same strain receiving control rat IgG [33]. Mechanistic analyses revealed that anti-CD137 antibody clone 2A significantly increased the number of activated CD8^+^ T cells which augmented the production of IFN-γ. Concomitant treatment with 2A and anti-IFN-γ led to normalization of DNTC, B cell and CD11b^+^Gr-1^+^ macrophages/granulocyte counts, suggesting that IFN-γ which was produced by the activated CD8^+^ T cells upon 2A treatment is crucial for reducing the numbers of pathogenic DNTC and autoantibody-producing B cells through the IFN-γ activated CD11b^+^Gr-1^+^ macrophages/granulocytes. Additionally, 2A treatment enhanced apoptosis of pathogenic DNTC as evidenced by the reduction in the proportion of annexin V^+^CD69^+^DNTC [33]. Lastly, an attempt to study sCD137 level across various strains of lupus-prone mice including NZB x NZW, BALB/c MRL-Faslpr/J, C57BL/6 MRL-Faslpr/J, C57BL/6 lpr and C57BL/6/gld mice revealed that sCD137 levels were higher than those of B6 wild type (WT) and BALB/c mice [35]. In addition, upon stimulation of splenic T cells with anti-CD3 and ConA, sCD137 levels were found to be increased in all mice in a non-strain specific manner [35]. In the quest for the functional characteristics of sCD137 in SLE, sCD137 was depleted in the splenocyte culture after which the cells were stimulated with agonistic CD137 antibody. Interestingly, the production of IL-2, IL-12 and IL-10 were found to be elevated upon CD137 stimulation in the absence of sCD137, suggesting that sCD137 functions as a regulator which blocks the interaction between CD137 expressed on activated T cells and dendritic cells and CD137L on other splenocytes, leading to reduction of IL-10 and most prominently of IL-12 [35]. Unfortunately, the therapeutic potential of sCD137 was not addressed in the study.

### 3.2. Human Studies

Studies of the CD137-CD137L system in human SLE are exceedingly scarce. In an attempt to address whether sCD137 and sCD137L are altered in patients with rheumatoid arthritis (RA), cellular expression and serum levels of sCD137 and sCD137L were demonstrated to be increased in patients with RA, SLE and Behcet’s disease compared to healthy subjects [7,36]. While in patients with RA serum sCD137 and sCD137L levels were significantly correlated and elevated in patients with higher RA disease activity, it was not addressed whether sCD137 and sCD137L behaved similarly in patients in SLE [36]. In addition, treatment with immunosuppressive agents was associated with sCD137 and sCD137L reductions in RA patients. However, the potential use of sCD137 and sCD137L levels in monitoring response in SLE patients was not addressed in the study [36].

Summarizing the observations in murine and human studies [32,33,34,35,36], blockade of the CD137-CD137L co-stimulation system by deleting the *CD137* gene leads to more severe SLE phenotypes including immune-mediated glomerulonephritis, profound lymphadenopathy and splenomegaly and dermatitis as a result of increased autoreactive pathogenic CD4^+^ T cells and functional B220^+^CD5^-^ B cells, leading to subsequent autoantibody production [37,38]. On the other hand, enhancing CD137 signalling via stimulation of CD137 by agonistic anti-CD137 antibodies enhances the Th1 polarization of T cells, and augments the production of IFN-γ by activation of CD8^+^ T cells, leading to apoptosis of autoreactive B cells and DNTC which are potent drivers of proinflammatory responses in SLE. While sCD137 level is increased in lupus-prone mice compared to WT mice, depletion of sCD137 prior to CD137 stimulation up-regulates the splenic production of IL-10 and IL-12 in lupus-prone mice, suggesting the regulatory role of sCD137 in counteracting an overwhelming pro-inflammatory immune response. Concurring with the murine models, cellular and serum expressions of sCD137 and sCD137L are increased in patients with SLE. Unfortunately, the immunological and clinical significance of sCD137 and sCD137L has not been addressed in SLE patients. Table 1 summarizes all the studies and their major findings of CD137 research in SLE in animal models and human disease. Despite our efforts in investigating the physiology and pathophysiology of the CD137-CD137L co-stimulation system in SLE for the past two decades, knowledge gaps remain. Thus far, we have substantial knowledge as to how the clinical and immunological phenotypes of SLE alter when CD137 signalling is manipulated. It is imperative to study CD137L signalling in SLE, with an aim to garner a better understanding regarding how the CD137-CD137L co-stimulation system works in SLE before proceeding to the next step of investigating the clinical safety and therapeutic efficacy of manipulating the CD137-CD137L system in patients with SLE.

## 4. What Is the Impact of Knocking out the *CD137L* Gene on SLE?

The understanding of the impact of the CD137-CD137L axis is incomplete if manipulation on the CD137L signalling is not investigated since immune deviation and subsequent phenotypic alterations are unpredictable if CD137L signalling is altered in SLE. As such, two hypothetical outcomes were proposed. First, ligating CD137L promotes adherence, differentiation and maturation of DCs and differentiation of B cells, which potentially worsen SLE. In addition, CD137L complexes with toll-like receptor (TLR) and enhances TLR signalling which is implicated in the pathogenesis of SLE [39]. Second, and on the contrary, the lack of CD137L abrogates the interaction between CD137 and CD137L which dampens Th1/Tc1 responses and manifests more severe lupus as described for CD137-deficient MRL-Fas^lpr^ mice [32]. In order to answer this critical question, our group has recently investigated the impact of the absence of the *CD137L* gene (and hence CD137L and CD137 signalling) on the phenotypic and immunological alterations in C57BL/6^lpr−/−^ (B6.lpr) mice [40]. B6.lpr mice were crossed to C57BL/6^CD137L−/−^ mice to obtain the CD137L-deficient B6.lpr (double knock out (DKO)) mice. The DKO mice were studied for their survival, immunological alterations and the extent of inflammation of major organs including the skin, kidneys and the brains, which were compared to those of B6.lpr and B6.WT mice. After observation for 22 months involving 226 DKO and 137 B6.lpr mice, significantly more frequent proliferative glomerulonephritis (33% versus 7.9%, *p* = 0.005), larger skin lesions and shorter survival (median survival: 44 weeks versus 74 weeks) were observed in the DKO as compared to the B6.lpr counterpart. Conversely, the absence of CD137L appears to protect the B6.lpr mice from structural and functional cerebral damage. In the DKO mice, we found that microglial activation (denoted by the number of Iba-1^+^ cells) and demyelination (studied by staining with 1% Luxol fast blue and 0.2% Cresyl violet acetate) in the cerebral cortex, hippocampus, thalamus and hypothalamus were significantly less pronounced than those of the B6.lpr and B6.WT mice [40]. The cerebral function was evaluated as evidenced by an electrophysiological study of hippocampal slices of the DKO, B6.lpr and B6.WT mice with respect to field excitatory post-synaptic potentials and late long-term potentiation (late-LTP) of CA1 apical dendrites which gauged long-term synaptic plasticity and memory. In response to continuous theta-burst stimulation at CA1 of the hippocampus for 180 min, late-LTP amongst the DKO mice was significantly higher than that of the B6.lpr mice although it was lower than that of B6.WT mice, suggesting that the absence of CD137L signalling reduced damage to long-term synaptic plasticity and partially reversed memory impairment in the B6.lpr mice [40].

**Figure 2:**
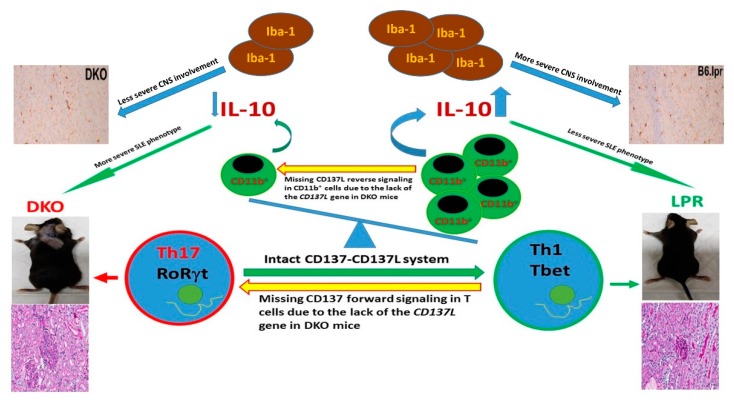
A diagram depicting the potential mechanisms related to the more severe SLE phenotype in the double knock out (DKO) mice than in B6.lpr mice. Interruption of the CD137-CD137L system by knocking out the *CD137L* gene in the DKO mice leads to a higher proportion of splenic Th17 cells as a result of missing CD137 forward signalling. A lower proportion of CD11b^+^ cells which produce IL-10 is likely due to missing CD137L reverse signalling, and a lower serum IL-10 level in the DKO mice is likely the result of a lower proportion of CD11b^+^ cells in the DKO mice, accompanied by a lower tendency to express IL-10. These three factors potentially promote the more severe SLE phenotype in the DKO than in the B6.lpr mice. Less activated lba-1^+^ microglia, on the contrary, leads to less severe pathology in the central nervous system.

Comprehensive mechanistic studies were of paramount importance to explain the discrepant impact of CD137L on the renal and brain tissues in the B6.lpr mice. While the frequencies of splenic B cells, T cells (CD4^+^, CD8^+^ and DNTC) and myeloid cells (granulocytes, monocytes and conventional DCs) did not differ between the DKO and B6.lpr mice as studied by polychromatic flow cytometric analyses, activated T cells, particularly those of the CD8^+^ compartment (CD3^+^CD8^+^CD69^+^), were significantly less frequent in DKO than in B6.lpr mice due to the absence of CD137-CD137L engagement. When different subsets of CD4^+^ T cells were further evaluated, the Th17 cells (CD3^+^CD4^+^RORγt^+^), which are potent drivers of SLE-related inflammation [41], were found to be significantly more frequent in the DKO than in the B6.lpr and B6.WT mice. Although overall intracellular IL-10 and IL-17 expression by T cells were not affected in the absence of CD137L, there were fewer IL-10-producing myeloid cells (CD11b^+^IL-10^+^) in the DKO mice, leading to lower serum IL-10 levels than in B6.lpr and B6.WT mice. Collectively, the absence of CD137L induces an immune deviation of CD4^+^ T cells by polarizing them towards the Th17 phenotype and reduces IL-10-producing CD11b^+^ cells and hence serum anti-inflammatory IL-10 levels, resulting in more severe lupus-related glomerulonephritis and dermatitis [42,43]. Notably, the absence of CD137L signalling in the DKO mice led to reduced activation of the microglia cells (myeloid in origin), reduced inflammatory cell infiltrates in the brain and cerebral demyelination, thus preserving cerebral function by protecting long-term synaptic plasticity in the DKO mice [40]. Figure 2 summarizes the mechanism as to how disruption of CD137-CD137L co-stimulation system leads to aggravation of SLE.

## 5. Conclusions and Future Direction

Costimulatory receptor–ligand systems play important pathogenic roles in SLE. While a few major translational and clinical trials have demonstrated therapeutic potential for SLE by blocking co-stimulation systems involving CTLA-4, B7 and CD40L [44], a major therapeutic breakthrough has yet to be achieved. The major culprits are our poor understanding of the immunological and signalling behaviour involved in co-stimulation and the lack of meticulous and educated patient selection strategies in previous clinical trials. The interesting yet challenging observations we have experienced while addressing the pathophysiology and therapeutic manipulation of the CD137-CD137L co-stimulation system in SLE, have let us appreciate more thoroughly the complexity of the co-stimulation system in mediating the pathophysiology of SLE. Based on our current understanding of the differential effects of CD137-CD137L manipulation in major organ manifestation in SLE, the next sensible step would be an evaluation of the clinical safety and efficacy of the therapeutic CD137 and/or CD137L manipulation in different subsets of SLE patients in accordance to their clinical manifestations. For example, in designing a clinical trial to manage patients with lupus glomerulonephritis, including CD137 stimulation with an agonistic anti-CD137 antibody would be a reasonable and educated approach. On the other hand, antagonistic anti-CD137L antibody should be the assessment option for patients with neuropsychiatric SLE.

Beyond their therapeutic potential, the CD137-CD137L system should also be further evaluated for its potential as a disease biomarker of SLE. For example, while serum sCD137 and sCD137L levels were shown to correlate with the disease activity of RA [36], it requires further investigation whether serum sCD137 and/or sCD137L are reliable markers of SLE disease activity. Likewise, how T cell expression of CD137 alters with clinical disease activity of SLE can be evaluated. In addition, the *CD137* and *CD137L* genes can also be evaluated for their potential as biomarkers of SLE. By adopting the next-generation sequencing technique, our centre is currently evaluating the impact of *CD137* and *CD137L* gene polymorphisms on clinical manifestations and treatment responses in our lupus cohort.

## Figures and Tables

**Table 1:** Studies on CD137 manipulation in animal and human systemic lupus erythematosus (SLE).

Mouse Model/Human (First Author, Year)	Intervention	Phenotypic Changes	CD4	CD8	DNTC or B Cells	AutoAb	Treg	sCD137
MRL^lpr^ (Vinay et al., 2007)	Knocking out CD137 gene	Exacerbation of SLE, ↓survival	↑	↔	↑/↑ in function	↑	ND	ND
MRL^lpr^ (Sun et al., 2002)	Agonistic anti-CD137Ab	Amelioration of SLE, ↑survival	↓	↑	↓/↓	↓	ND	ND
NZB/W _F1_ (Foell et al., 2003)	Agonistic anti-CD137Ab	Amelioration of SLE, ↑survival	↔	↔	ND/↔	↓	↑	ND
BALB/c and B6^/lpr^ or gld ^−/−^ (Shao et al., 2008)	nil	ND	ND	ND	ND	ND	ND	Increase in lpr^−/−^ and gld^−/−^ mice regardless of background
B6.MRL^lpr^ (Mak et al., 2019)	Knocking out CD137L gene	Exacerbation of nephritis & dermatitis, ↓survival but amelioration of CNS inflammation	↔, but ↑Th17	↓activated CD8^+^ T cells	↔	ND	↔	ND
Human (Jung et al., 2004)	nil	ND	ND	ND	ND	ND	ND	sCD137 and sCD137L increase in SLE and they correlate with each other

Abbreviations: CD, cluster of differentiation; DNTC, double negative T cells, Ab; antibody, Treg, regulatory T cells; sCD137, soluble CD137; ND, not done; CD137L, CD137 ligand; sCD137L, soluble CD137ligand. ↑ increase; 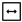 unchanged; ↓ decrease.
